# Supplementary material for: PAN-cancer analysis of S-phase enriched lncRNAs identifies oncogenic drivers and biomarkers
Source: Nat Commun. 2018 Feb 28;9:883. doi: 10.1038/s41467-018-03265-1 (PMC5830406; doi:10.1038/s41467-018-03265-1)
Supplement: Supplementary file 1 — Supplementary Information [file 41467_2018_3265_MOESM1_ESM.pdf]

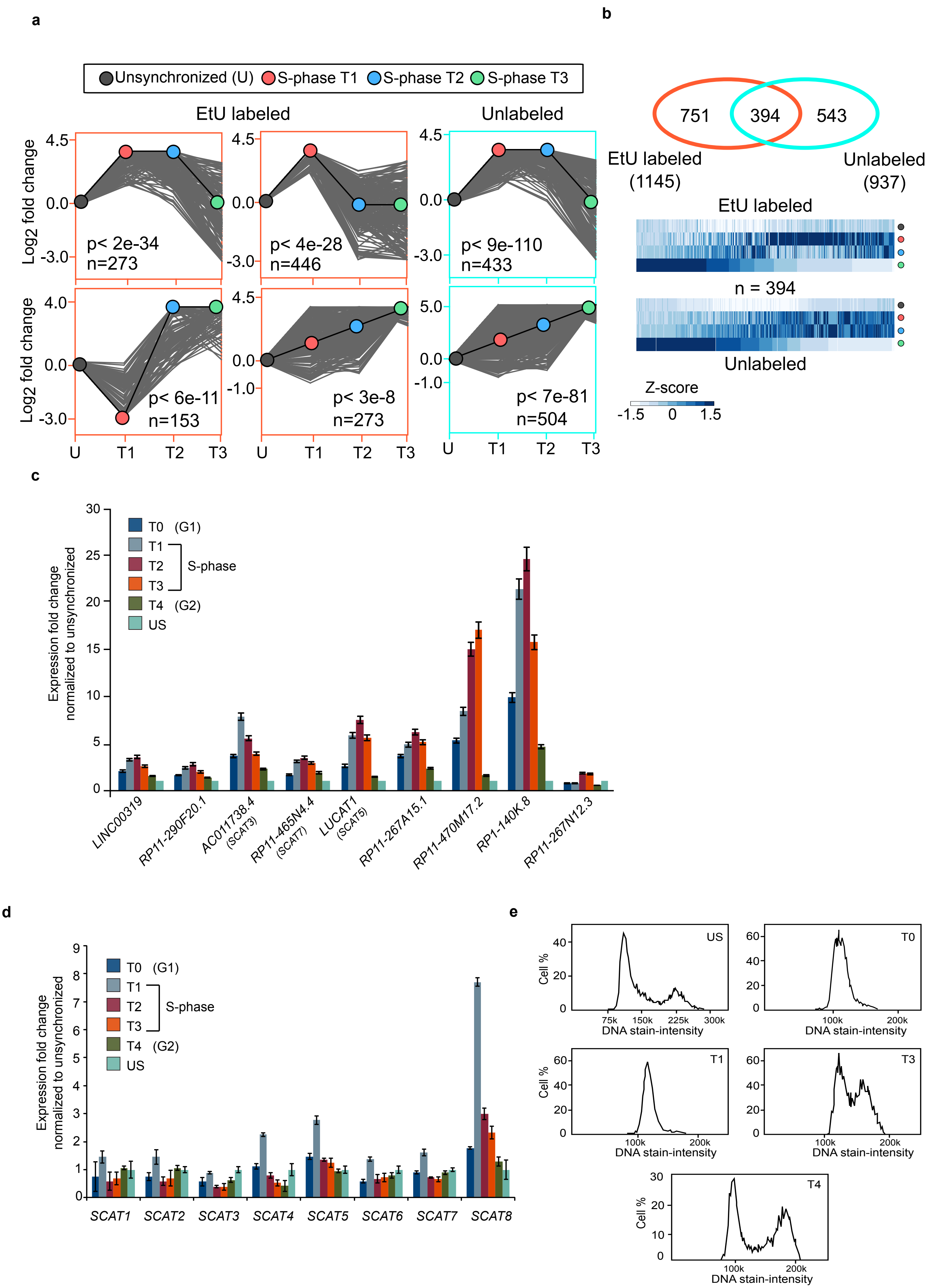

a
Unsynchronized (U) S-phase T1 S-phase T2 S-phase T3
EtU labeled
Unlabeled
Log2 fold change
4.5
0.0
-3.0
p< 2e-34
n=273
p< 4e-28
n=446
p< 9e-110
n=433
4.0
p< 6e-11
n=153
-1.0
p< 3e-8
n=273
5.0
p< 7e-81
n=504
U
T1
T2
T3
b
751
394
543
EtU labeled
(1145)
Unlabeled
(937)
EtU labeled
n = 394
Unlabeled
Z-score
-1.5
0
1.5
c
Expression fold change
normalized to unsynchronized
T0 (G1)
T1
T2
T3
S-phase
T4 (G2)
US
LINC00319
RP11-290F20.1
AC011738.4
(SCAT3)
RP11-465N4.4
(SCAT7)
LUCAT1
(SCAT5)
RP11-267A15.1
RP11-470M17.2
RP1-140K.8
RP11-267N12.3
d
Expression fold change
normalized to unsynchronized
T0 (G1)
T1
T2
T3
S-phase
T4 (G2)
US
SCAT1
SCAT2
SCAT3
SCAT4
SCAT5
SCAT6
SCAT7
SCAT8
e
Cell %
DNA stain-intensity
US
T0
T1
T3
T4
75k
150k
225k
300k
100k
200k

Supplementary Fig. 1. Temporal expression of lncRNAs across S-phase time points. **a** STEM analysis showing temporal expression of cell cycle associated lncRNAs in EtU labeled and unlabeled samples. The p-values are obtained using permutation tests from STEM clustering. **b** Venn diagram indicates the number of temporally expressed S-phase lncRNAs in EtU labeled and unlabeled samples (upper panel). The Heatmap (lower panel) shows the expression patterns of 394 common S-phase lncRNAs detected in EtU labeled and unlabeled samples at different time points. **c** Real-time qPCR validation using nascent capture assay for randomly selected S-phase lncRNAs in synchronized EtU labeled samples collected at different time points of the cell cycle. The data are expressed as expression fold change over the unsynchronized (US) sample. **d** Real-time qPCR validation of selected S-phase lncRNAs using total RNA collected at different time points of the cell cycle in serum-starved cells. The data are expressed as expression fold change over the unsynchronized (US) sample. **e** Flow cytometry histogram showing the cell cycle profiles of HeLa cells synchronized with serum-free method at different time points.

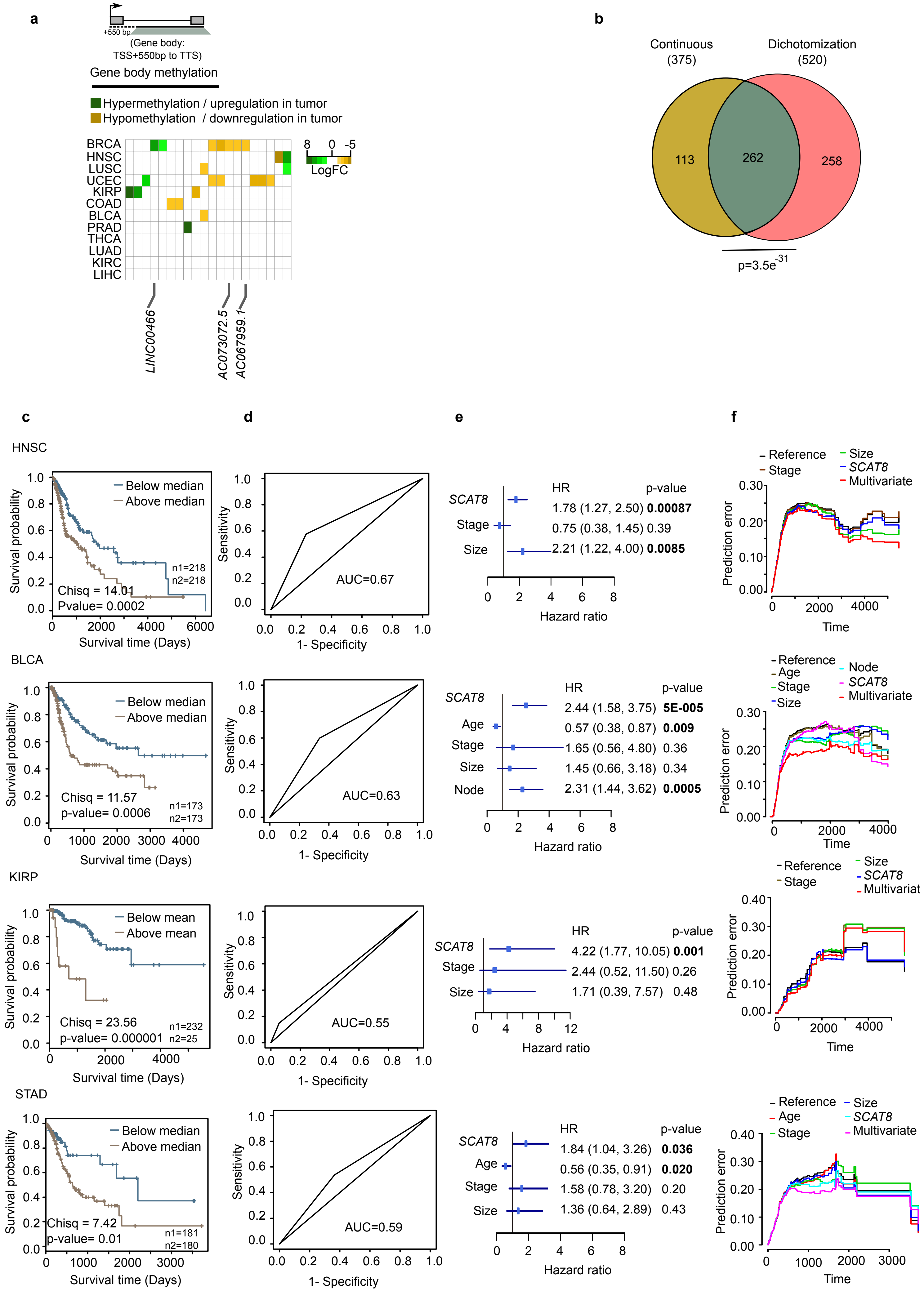

a
+550 bp
(Gene body: TSS+550bp to TTS)
Gene body methylation
Hypermethylation / upregulation in tumor
Hypomethylation / downregulation in tumor
BRCA
HNSC
LUSC
UCEC
KIRP
COAD
BLCA
PRAD
THCA
LUAD
KIRC
LIHC
8 0 -5
LogFC
LINC00466
AC073072.5
AC067959.1
b
Continuous (375)
Dichotomization (520)
113
262
258
p=3.5e-31
c
d
e
f
HNSC
Below median
Above median
n1=218
n2=218
Chisq = 14.01
Pvalue= 0.0002
Survival probability
Survival time (Days)
AUC=0.67
Sensitivity
1- Specificity
SCAT8
Stage
Size
HR
p-value
1.78 (1.27, 2.50) 0.00087
0.75 (0.38, 1.45) 0.39
2.21 (1.22, 4.00) 0.0085
Hazard ratio
Reference
Stage
Size
SCAT8
Multivariate
Prediction error
Time
BLCA
Chisq = 11.57
p-value= 0.0006
n1=173
n2=173
AUC=0.63
SCAT8
Age
Stage
Size
Node
2.44 (1.58, 3.75) 5E-005
0.57 (0.38, 0.87) 0.009
1.65 (0.56, 4.80) 0.36
1.45 (0.66, 3.18) 0.34
2.31 (1.44, 3.62) 0.0005
KIRP
Below mean
Above mean
Chisq = 23.56
p-value= 0.000001
n1=232
n2=25
AUC=0.55
4.22 (1.77, 10.05) 0.001
2.44 (0.52, 11.50) 0.26
1.71 (0.39, 7.57) 0.48
Multivariat
STAD
Chisq = 7.42
p-value= 0.01
n1=181
n2=180
AUC=0.59
1.84 (1.04, 3.26) 0.036
0.56 (0.35, 0.91) 0.020
1.58 (0.78, 3.20) 0.20
1.36 (0.64, 2.89) 0.43

Supplementary Fig. 2. Epigenetic alteration and clinical investigation of SCATs across multiple cancer types. **a** Heatmap shows S-phase lncRNAs having positive correlation of gene body methylation with the differential expression in the corresponding cancer. Hypomethylated gene body with lower expression and hypermethylation associated with higher expression compared to normal. The highlighted lncRNAs have more than 100 patients (samples) supporting the methylation status in the corresponding cancer. **b** Venn diagram shows a significant overlap between clinically-relevant S-phase lncRNAs derived from continuous and dichotomization-based approaches. The statistical significance was calculated using Fisher's exact test. **c** Kaplan-Meier plots of *SCAT8* indicating overall survival of patients in HNSC, BLCA, KIRP and STAD cancer types. The higher expression of the *SCAT8* predicts poor overall survival outcome. The statistical significance was calculated using logrank test. **d** ROC curves indicating the effectiveness of the prognostic index in predicting the survival probability outcome in each corresponding cancer as measured by area under the curve (AUC). **e** Multivariate models (Cox proportional hazard analysis): The impact of *SCAT8* and significant clinical covariates on overall survival probability of patients in HNSC, BLCA, KIRP and STAD cancer types. Estimated hazard ratios of *SCAT8* and pathologic tumor stage and size in HNSC; *SCAT8*, pathologic stage, age at diagnosis, size and node in BLCA; *SCAT58* and pathologic tumor stage and size in KIRP; *SCAT124*, age at diagnosis and pathologic tumor stage and size in STAD. The statistical significance was calculated using Wald test. **f** Brier prediction curves depicting the performance of a multivariate model compared to a reference model.

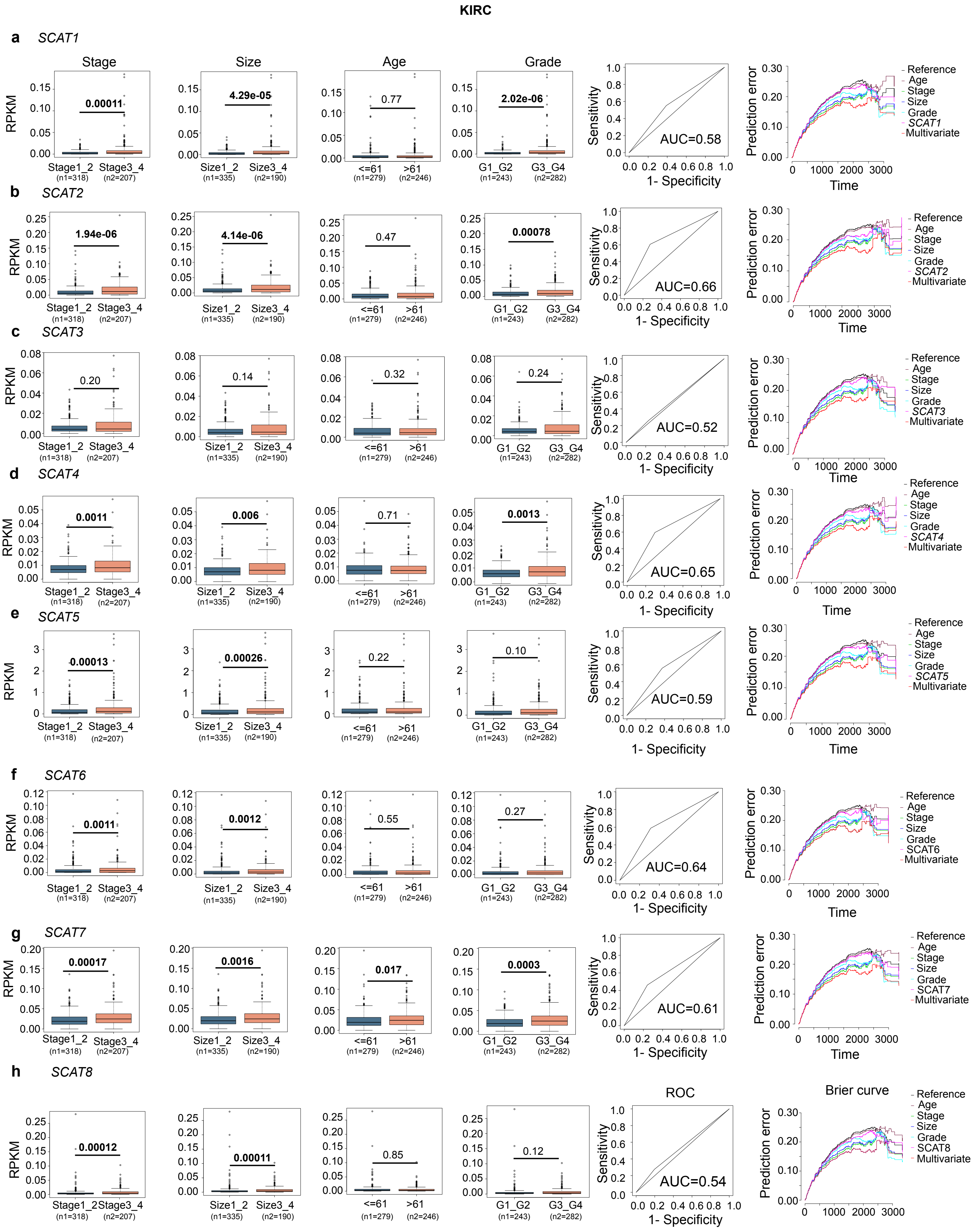
KIRC
a SCAT1
Stage
Size
Age
Grade
RPKM
0.00011
4.29e-05
0.77
2.02e-06
Stage1_2 (n1=318)
Stage3_4 (n2=207)
Size1_2 (n1=335)
Size3_4 (n2=190)
<=61 (n1=279)
>61 (n2=246)
G1_G2 (n1=243)
G3_G4 (n2=282)
Sensitivity
1- Specificity
AUC=0.58
Prediction error
Time
Reference
Age
Stage
Size
Grade
SCAT1
Multivariate
b SCAT2
1.94e-06
4.14e-06
0.47
0.00078
AUC=0.66
c SCAT3
0.20
0.14
0.32
0.24
AUC=0.52
d SCAT4
0.0011
0.006
0.71
0.0013
AUC=0.65
e SCAT5
0.00013
0.00026
0.22
0.10
AUC=0.59
f SCAT6
0.0011
0.0012
0.55
0.27
AUC=0.64
g SCAT7
0.00017
0.0016
0.017
0.0003
AUC=0.61
h SCAT8
0.00012
0.00011
0.85
0.12
ROC
Brier curve
AUC=0.54

i

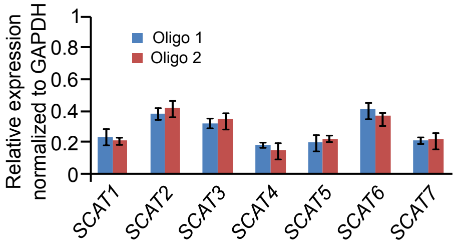
Relative expression normalized to GAPDH
1
0.8
0.6
0.4
0.2
0
Oligo 1
Oligo 2
SCAT1
SCAT2
SCAT3
SCAT4
SCAT5
SCAT6
SCAT7

j

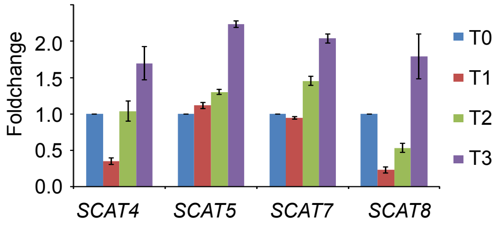
Foldchange
2.0
1.5
1.0
0.5
0.0
SCAT4
SCAT5
SCAT7
SCAT8
T0
T1
T2
T3

k

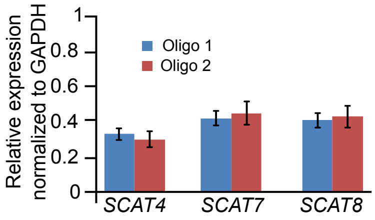
Relative expression normalized to GAPDH
1
0.8
0.6
0.4
0.2
0
Oligo 1
Oligo 2
SCAT4
SCAT7
SCAT8

Supplementary Fig. 3. Top SCATs acting as independent prognostic markers in KIRC. **a-h** Boxplots showing the significant association between the expression levels of different SCATs and other clinical covariates in KIRC (left panels). The ROC curves (middle panels) indicate the effectiveness of the prognostic index in predicting the survival probability outcome of KIRC patients. Brier prediction curves (right panels) depict the performance of a multivariate models compared to a reference model in KIRC. **i** Real-time qPCR validation of the KD efficiency of *SCAT1−SCAT7* in HeLa cells using two independent oligonucleotides (LNAs or siRNAs). **j** Real-time qPCR validation of the selected SCATs using total RNA isolated from drug-free synchronized Caki-2 cells at different time points of the cell cycle. The data are expressed as fold change in expression over synchronized G1 cells. **k** Real-time qPCR validation of the KD efficiency of *SCAT4*, *SCAT7* and *SCAT8* in Caki-2 cells using two independent oligonucleotides (LNAs or siRNAs). Statistical significance shown in boxplots (**Supplementary Fig. 3a-h**) was derived using a two-tailed unpaired student's t-test or Wilcoxon Rank Sum test.

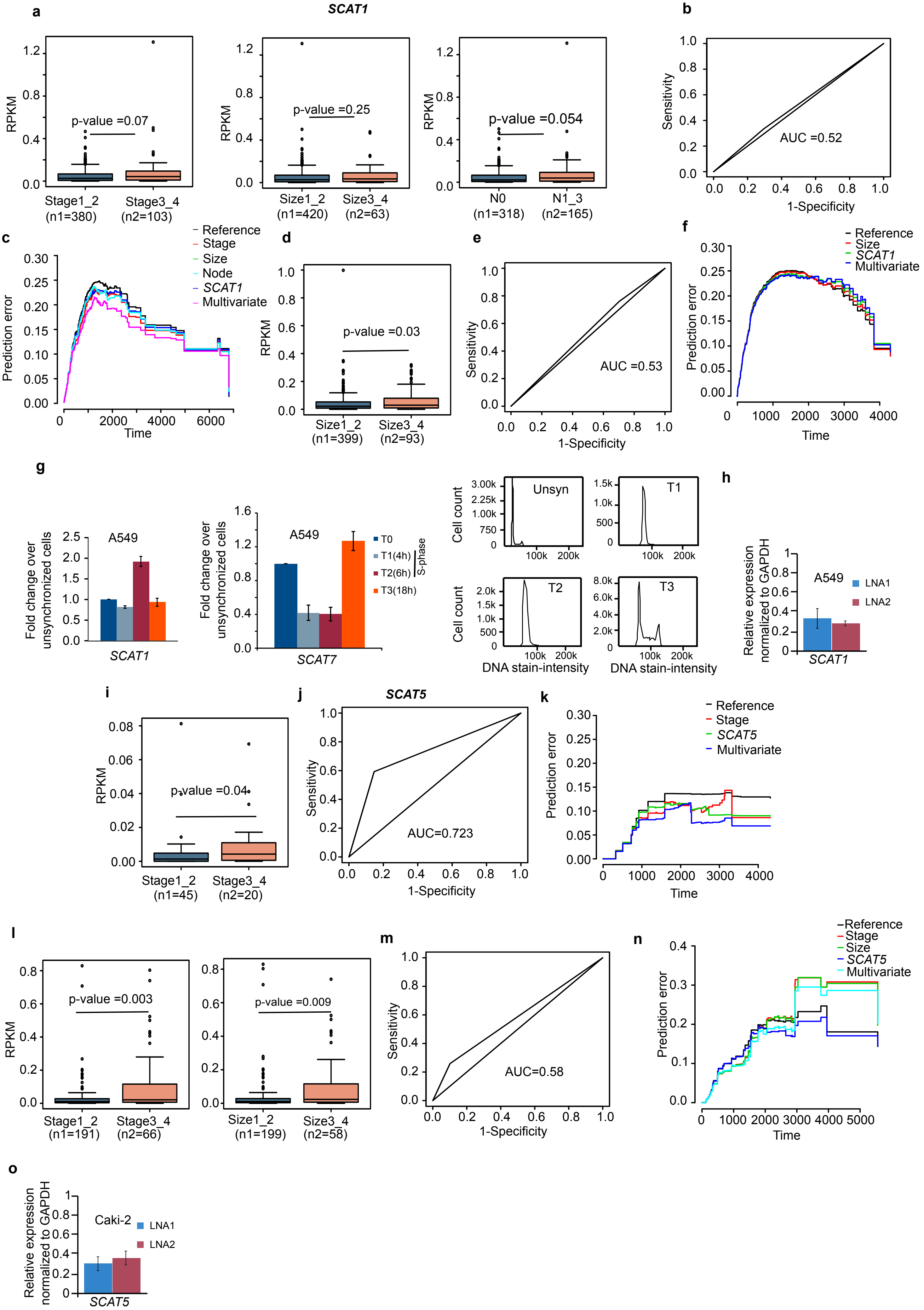
a
SCAT1
RPKM
p-value =0.07
Stage1_2 (n1=380)
Stage3_4 (n2=103)
p-value =0.25
Size1_2 (n1=420)
Size3_4 (n2=63)
p-value =0.054
N0 (n1=318)
N1_3 (n2=165)
b
Sensitivity
1-Specificity
AUC =0.52
c
Prediction error
Time
Reference
Stage
Size
Node
SCAT1
Multivariate
d
RPKM
p-value =0.03
Size1_2 (n1=399)
Size3_4 (n2=93)
e
Sensitivity
1-Specificity
AUC =0.53
f
Prediction error
Time
Reference
Size
SCAT1
Multivariate
g
Fold change over unsynchronized cells
A549
SCAT1
SCAT7
T0
T1(4h)
T2(6h)
T3(18h)
S-phase
Cell count
Unsyn
T1
T2
T3
DNA stain-intensity
h
Relative expression normalized to GAPDH
A549
LNA1
LNA2
SCAT1
i
RPKM
p-value =0.04
Stage1_2 (n1=45)
Stage3_4 (n2=20)
j
SCAT5
Sensitivity
1-Specificity
AUC=0.723
k
Prediction error
Time
Reference
Stage
SCAT5
Multivariate
l
RPKM
p-value =0.003
Stage1_2 (n1=191)
Stage3_4 (n2=66)
p-value =0.009
Size1_2 (n1=199)
Size3_4 (n2=58)
m
Sensitivity
1-Specificity
AUC=0.58
n
Prediction error
Time
Reference
Stage
Size
SCAT5
Multivariate
o
Relative expression normalized to GAPDH
Caki-2
LNA1
LNA2
SCAT5

Supplementary Fig. 4. Clinical investigation of *SCAT1* and *SCAT5* in kidney and lung-derived tumors, respectively. **a-c** Boxplots showing the significant association between the expression levels of *SCAT1* and clinical covariates; pathologic tumor stage, size and node in LUAD patients (**a**). ROC curve indicates the effectiveness of the prognostic index in predicting the survival probability (**b**). Brier prediction curves depicts the performance of a multivariate models compared to a reference model (**c**). **d-f** Boxplots showing the significant association between the expression levels of *SCAT1* and pathologic tumor size in LUSC patients (**d**). The ROC curve shows the effectiveness of the prognostic index in predicting the survival probability outcome of LUSC patients (**e**). Brier prediction curves depicts the performance of a multivariate models compared to a reference model (**f**). **g** Real-time qPCR validation of *SCAT1* and *SCAT7* using total RNA isolated from drug-free synchronized A549 cells at different time points of the cell cycle (left panel). The data are expressed as fold change in expression over unsynchronized cells. **h** Real-time qPCR validation of the knockdown efficiency of SCAT1 in A549 cells using two different LNAs. **i-k** Boxplots showing the significant association between the expression levels of *SCAT5* and pathologic tumor stage in KIRP patients (**i**). The ROC curve shows the effectiveness of the prognostic index in predicting the survival probability outcome of (**j**). Brier prediction curves depicts the performance of a multivariate models compared to a reference model (**k**). **l-n** Boxplots showing the significant association between the expression levels of *SCAT5* and pathologic tumor stage and size in KICH patients (**l**). The ROC curve shows the effectiveness of the prognostic index in predicting the survival probability outcome of (**m**). Brier prediction curves depicts the performance of a multivariate models compared to a reference model (**n**). **o** Real-time qPCR validation of the KD efficiency of *SCAT5* using two independent LNAs in Caki-2 cell lines. Statistical significance shown in boxplots (**Supplementary Fig. 4a, d, i and l**) was derived using a two-tailed unpaired student's t-test or Wilcoxon Rank Sum test.

a

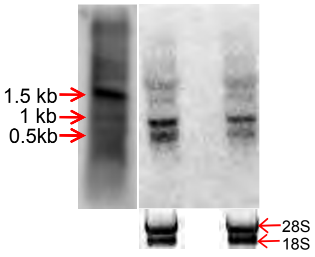

SCAT7 variants	CPAT_ probability	CPAT_ coding_label	CPC_score	CPC_status
RP11-465N4.4-001	0.061	no	-0.979	noncoding
RP11-465N4.4-002	0.104	no	-0.842	noncoding

b

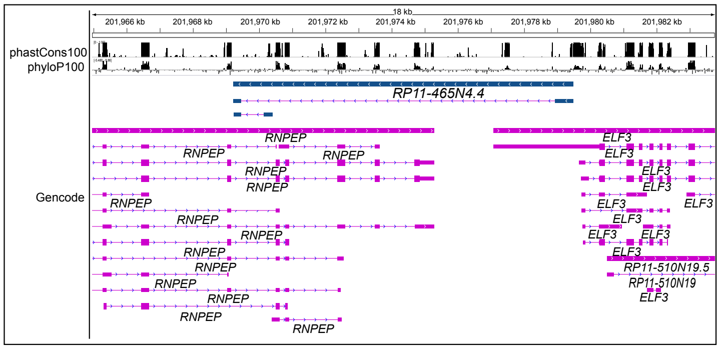

c
d
e
f
g
h
i

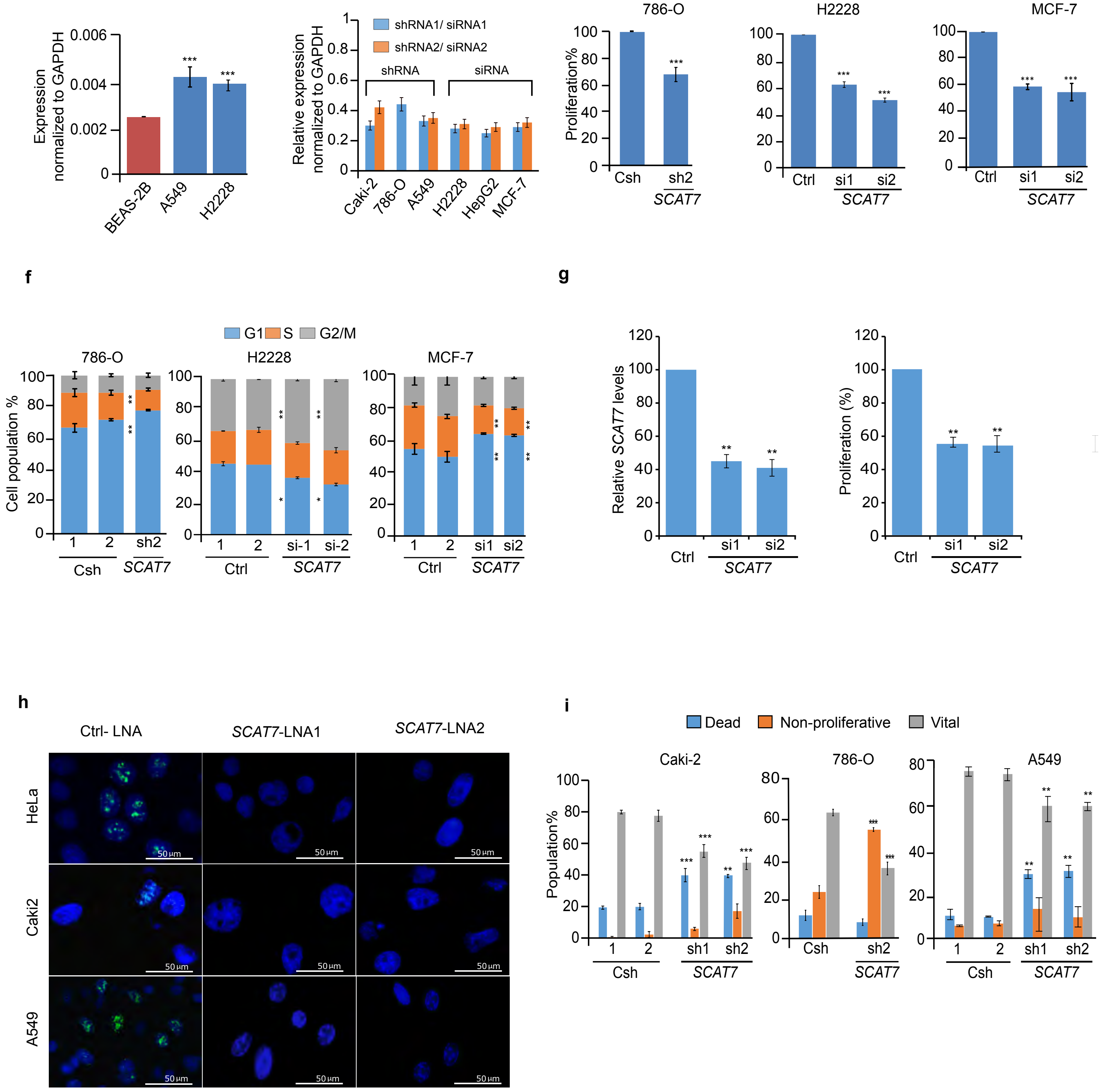

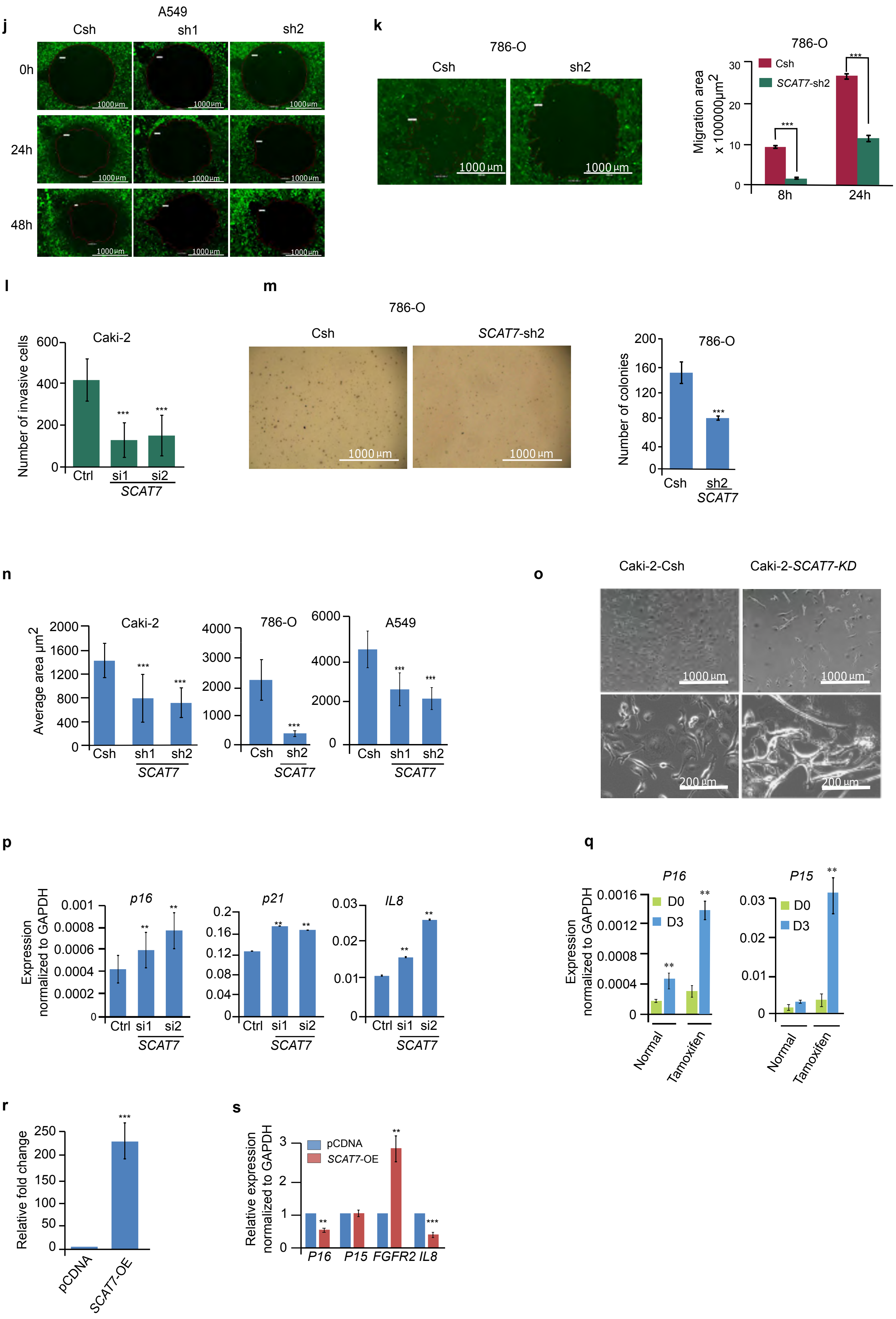

j
A549
Csh
sh1
sh2
0h
24h
48h
1000 μm
k
786-O
Csh
sh2
786-O
Migration area x 100000μm2
Csh
SCAT7-sh2
***
30
20
10
0
8h
24h
l
Caki-2
Number of invasive cells
600
400
200
0
***
Ctrl
si1
si2
SCAT7
m
786-O
Csh
SCAT7-sh2
1000 μm
786-O
Number of colonies
200
160
120
80
40
0
***
Csh
sh2
SCAT7
n
Caki-2
Average area μm2
2000
1600
1200
800
400
0
***
Csh
sh1
sh2
SCAT7
786-O
4000
3000
2000
1000
0
Csh
sh2
SCAT7
A549
6000
4000
2000
0
Csh
sh1
sh2
SCAT7
o
Caki-2-Csh
Caki-2-SCAT7-KD
1000 μm
200 μm
p
Expression normalized to GAPDH
p16
0.001
0.0008
0.0006
0.0004
0.0002
0
**
Ctrl
si1
si2
SCAT7
p21
0.2
0.16
0.12
0.08
0.04
0
IL8
0.03
0.02
0.01
0
q
Expression normalized to GAPDH
P16
0.0016
0.0012
0.0008
0.0004
0
D0
D3
Normal
Tamoxifen
P15
0.03
0.02
0.01
0
r
Relative fold change
250
200
150
100
50
0
***
pCDNA
SCAT7-OE
s
Relative expression normalized to GAPDH
pCDNA
SCAT7-OE
3
2
1
0
P16
P15
FGFR2
IL8

Supplementary Fig. 5. Functional analysis of *SCAT7* in renal, lung and liver cancers. **a** Northern blot showing the size of *SCAT7* variants in wild type and KD A549 cells (upper panel). The lower panel indicates the noncoding capacity of *SCAT7* variants using two different tools. **b** Sequence conservation analysis indicates no conserved homology of *SCAT7* sequence among vertebrates. **c** Real-time qPCR expression analysis of *SCAT7* in normal human lung cells (BEAS-2B) compared to A549 and H2228 lung cancer cell lines. **d** Real-time qPCR validation of the KD efficiency of *SCAT7* in multiple cell lines using shRNAs or siRNAs. The KD efficiency of each cell line was determined with respect to the corresponding negative control. **e** Proliferation capacity of 786-O, H2228 and MCF-7 cell lines as measured by MTT colorimetric assay post-silencing of *SCAT7* using shRNA or siRNAs. Data are represented as percentage in respect to cells transfected with negative control. **f** Cell cycle profiles of 786-O, H2228 and MCF-7 cells depleted with shRNA or two different siRNAs targeting *SCAT7*. Both 786-O and MCF-7 cell lines show significant accumulation at the G1 boundary with reduced DNA synthesis. H2228 KD cells show significant accumulation at G2/M phase with reduction of G1 phase. **g** Real-time qPCR validation of SCAT7 KD efficiency upon treatment with two SCAT7 siRNAs in HEK293 cells (left panel). Proliferation capacity of HEK293 cells measured by MTT colorimetric assay post-silencing of *SCAT7* using siRNAs. **h** EdU incorporation assay in wild HeLa, Caki-2 and A549 cells showing less incorporation of the fluorescently-labeled EdU analogue upon *SCAT7* KD. Scale bars, 50µm. **i** Assessment of cellular vitality of Caki-2, 786-O and A549 stable *SCAT7* KD cell lines using a mixture of acridine orange, DAPI and VB-48 stains. With respect to the corresponding control, all KD cells show areduction in population percentage of active proliferative cells with asignificant increase in percentages of non-proliferative or dead cells. **j** Migration assay of two GFP-labeled stable A549 *SCAT7* KD cell lines. GFP-labeled migrating cells were tracked at different time points. Different migration surfaces were quantified using an automated area estimation function. Scale bars, 1000µm. **k** Migration assay of GFP-labeled 786-O stable KD cells 24h post-seeding. The bar graph on the right panel shows the quantification of the migration surfaces of 786-O control and KD cells at two-time points. Scale bars, 1000µm. **l** Matrigel-transwell invasion assay for Caki-2 cells transfected with two different siRNAs. The number of invasive cells was

counted 48 h post-transfection. **m** Soft agar colony forming assay of 786-O stable KD cells. The bar graph represents the average number of colonies. Images were captured and quantified using an automated Z-stack function (left panel). Scale bars, 1000µm. **n** Estimation of the average surface area of individual colonies in different stable KD cells after 10 days of incubation in soft agar. **o** Deformed morphology of Caki-2 cells upon *SCAT7* depletion. Scale bars, 1000µm (upper panel) and 200 µm (lower panel). **p** Expression levels of *p16*, *p21*, and *IL8* in BJ-BRAF human fibroblast cells following transient silencing of *SCAT7* with two independent siRNAs. The expression values are normalized to GAPDH. **q** Real-time qPCR validation of the elevated expression of senescence-associated genes at day0 and day 3 post tamoxifen treatment. **r** Quantification of *SCAT7* expression level following transfection with *SCAT7*-overexpression vector. Data are expressed as relative fold change of expression compared to an empty control vector. **s** Gene expression quantification of senescence-associated genes upon over-expression of *SCAT7*. Expression values are normalized to GAPDH in comparison with cells transfected with empty vector. Statistical significance shown in **Supplementary Fig. 5c, e-g, i, k-n and p-s** were derived using a two-tailed unpaired student's t-test.

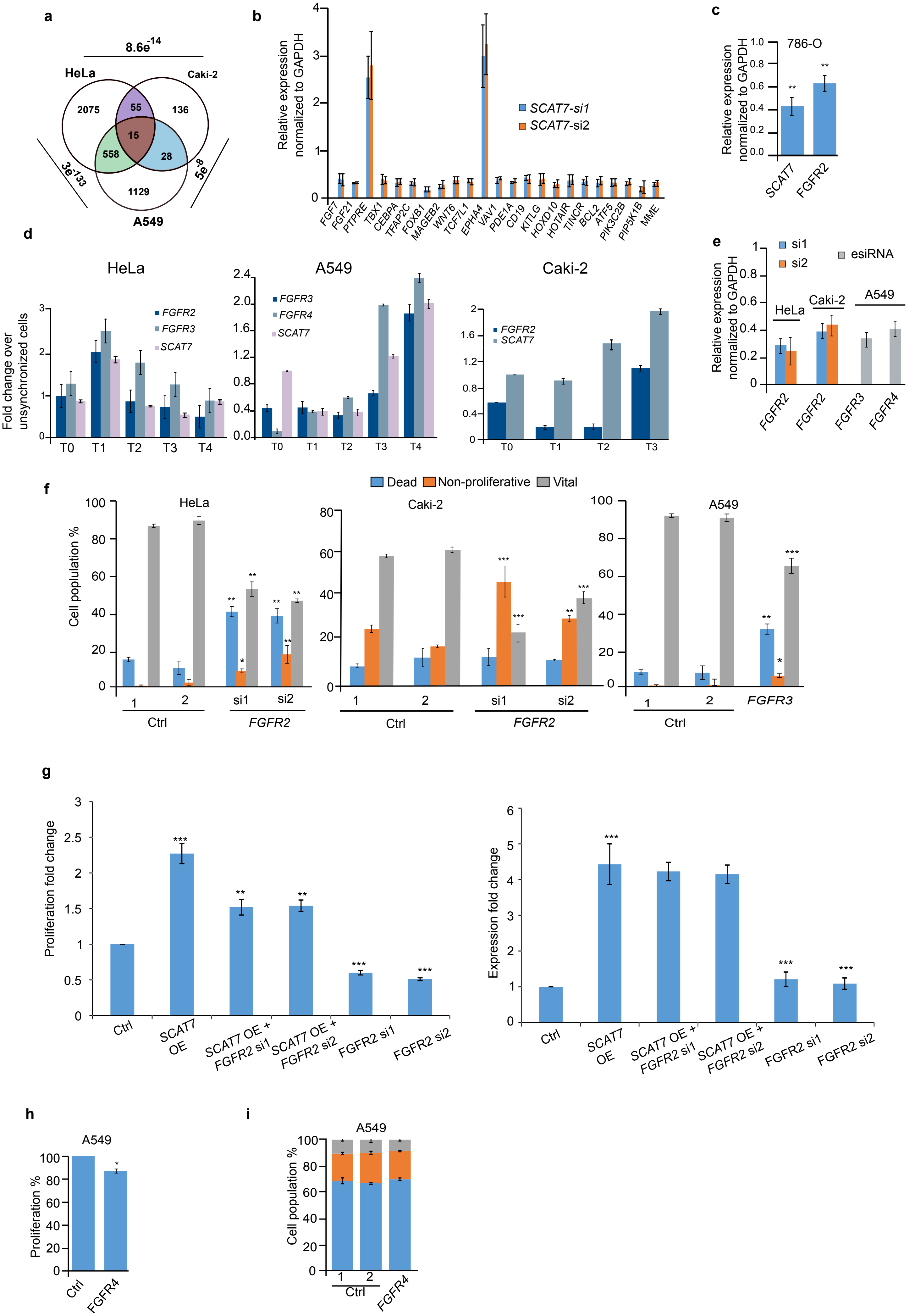
a
8.6e-14
HeLa
Caki-2
2075
55
136
15
558
28
1129
3e-133
5e-8
A549
b
Relative expression normalized to GAPDH
SCAT7-si1
SCAT7-si2
FGF7
FGF21
PTPRE
TBX1
CEBPA
TFAP2C
FOXB1
MAGEB2
WNT6
TCF7L1
EPHA4
VAV1
PDE1A
CD19
KITLG
HOXD10
HOTAIR
TINCR
BCL2
ATF5
PIK3C2B
PIP5K1B
MME
c
786-O
Relative expression normalized to GAPDH
SCAT7
FGFR2
d
HeLa
A549
Caki-2
Fold change over unsynchronized cells
FGFR2
FGFR3
SCAT7
FGFR4
T0
T1
T2
T3
T4
e
si1
si2
esiRNA
HeLa
Caki-2
A549
Relative expression normalized to GAPDH
FGFR2
FGFR2
FGFR3
FGFR4
f
Dead
Non-proliferative
Vital
HeLa
Caki-2
A549
Cell population %
1
2
si1
si2
Ctrl
FGFR2
FGFR3
g
Proliferation fold change
Expression fold change
Ctrl
SCAT7 OE
SCAT7 OE + FGFR2 si1
SCAT7 OE + FGFR2 si2
FGFR2 si1
FGFR2 si2
h
A549
Proliferation %
Ctrl
FGFR4
i
A549
Cell population %
1
2
Ctrl
FGFR4

Supplementary Fig. 6. Regulation of FGF signaling by *SCAT7*. **a** Venn diagram showing the number of differentially expressed genes upon *SCAT7* KD in different cell lines. The overlaps indicated the common differentially expressed genes between the cell lines. The statistical significance of each overlap was derived using Fisher's exact test and the p-values are indicated. **b** Real-time qPCR validation of *SCAT7* target genes obtained from RNA-seq data in HeLa KD cells. **c** Validation of the significant downregulation of *FGFR2* upon stable KD of *SCAT7* in 786-O cells. **d** Real-time qPCR validation of expression of different FGFR members in HeLa, A549 and Caki-2 cell lines using total RNA isolated from drug-free synchronized cells at different time points. **e** Real-time qPCR validation of the KD efficiency of different *FGFR* members in HeLa, Caki-2 and A549 cells. Two different siRNAs targeting *FGFR2* were used in HeLa and Caki-2 cells. Silencing of *FGFR3* and *FGFR4* was performed with esiRNAs. The KD efficiency of each transfection was assessed with respect to the corresponding control. **f** Assessment of cellular vitality of HeLa, Caki-2 and A549 cells using a mixture of acridine orange, DAPI and VB-48 stains upon depletion of *FGFR2* or *FGFR3*. Withrespect to the corresponding control, all KD cells show areduction in population percentage of active proliferative cells. **g** Effect on cell proliferation upon *FGFR2* knockdown in cells overexpressing *SCAT7* (left panel). The significance of *SCAT7* overexpression and *FGFR2* silencing is presented with respect to the control (right panel). **h** Estimation of the proliferation capacity of A549 silenced with esiRNAs targeting *FGFR4*. **i** Cell cycle profiling of A549 cells 48h post-silencing of *FGFR4* shows insignificant effect on cell cycle progression.

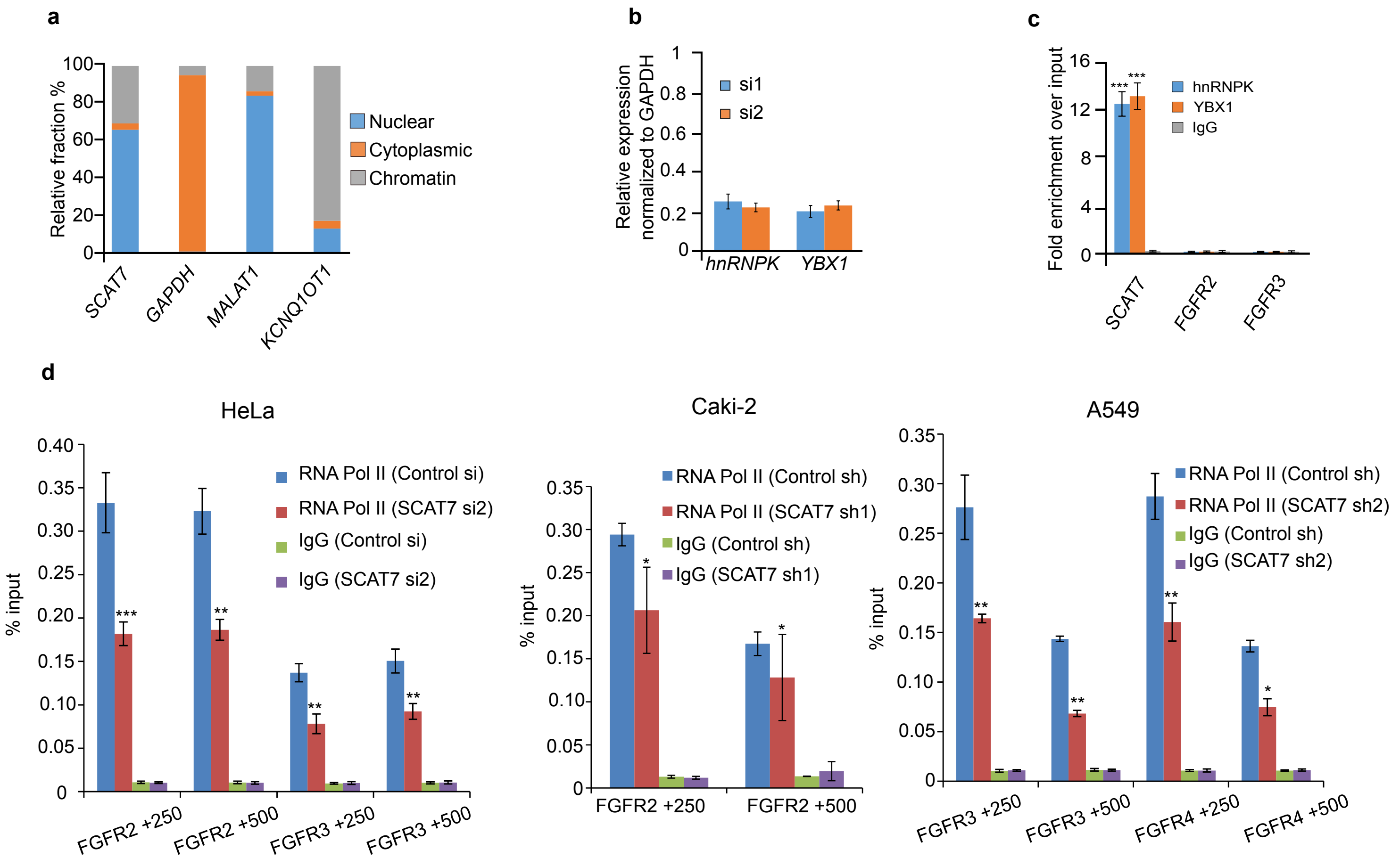

a
Relative fraction %
100
80
60
40
20
0
Nuclear
Cytoplasmic
Chromatin
SCAT7
GAPDH
MALAT1
KCNQ1OT1
b
Relative expression normalized to GAPDH
1
0.8
0.6
0.4
0.2
0
si1
si2
hnRNPK
YBX1
c
Fold enrichment over input
16
12
8
4
0
***
***
hnRNPK
YBX1
IgG
SCAT7
FGFR2
FGFR3
d
HeLa
% input
0.40
0.35
0.30
0.25
0.20
0.15
0.10
0.05
0
RNA Pol II (Control si)
RNA Pol II (SCAT7 si2)
IgG (Control si)
IgG (SCAT7 si2)
***
**
**
**
FGFR2 +250
FGFR2 +500
FGFR3 +250
FGFR3 +500
Caki-2
% input
0.35
0.30
0.25
0.20
0.15
0.10
0.05
0
RNA Pol II (Control sh)
RNA Pol II (SCAT7 sh1)
IgG (Control sh)
IgG (SCAT7 sh1)
*
*
FGFR2 +250
FGFR2 +500
A549
% input
0.35
0.30
0.25
0.20
0.15
0.10
0.05
0
RNA Pol II (Control sh)
RNA Pol II (SCAT7 sh2)
IgG (Control sh)
IgG (SCAT7 sh2)
**
**
**
*
FGFR3 +250
FGFR3 +500
FGFR4 +250
FGFR4 +500

Supplementary Fig. 7. Subcellular localization of *SCAT7*, knockdown efficiency of hnRNPK and YBX1 and Pull down of SCAT7, hnRNPK, YBX1 in A549 and Caki-2 cells. **a** Estimation of the relative enrichment of *SCAT7* in different cellular compartments. *GAPDH*, *MALAT1* and *KCNQ1OT1* were used as positive controls for the enrichment in cytoplasmic, nucleoplasmic and chromatin compartments, respectively. Equal amounts of the input RNA were used to estimate the relative enrichment in qPCR. **b** Validation of the KD efficiency of *hnRNPK* and *YBX1* using qPCR. **c** RIP assay with hnRNPK or YBX1 antibody followed by qPCR showing the relative enrichment of *SCAT7*, *FGFR2* and *FGFR3* mRNAs. **d** Assesment of RNA Pol II occupancy in the coding regions (250 and 500 bp downstream to TSS) of different FGFRs upon silencing of SCAT7 in HeLa, A549 and Caki-2 cells. Data in **(c)** and **(d)** are plotted as Mean ± SD from two biological replicates. *** P≤ 0.001, ** P≤0.01, * P≤0.05 (two-tailed unpaired student's t-test).

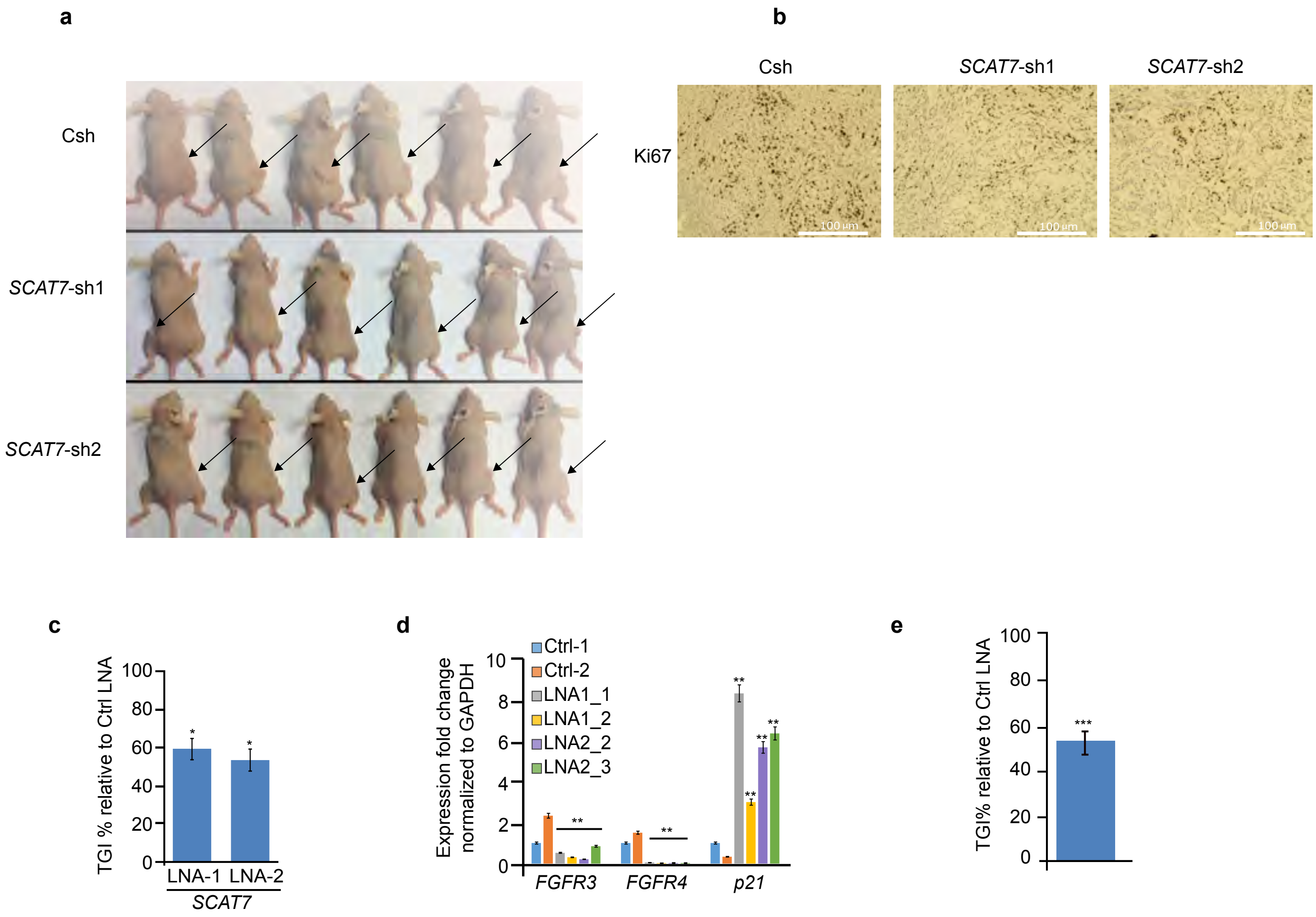
a
Csh
SCAT7-sh1
SCAT7-sh2
b
Csh
SCAT7-sh1
SCAT7-sh2
Ki67
100 µm
c
TGI % relative to Ctrl LNA
100
80
60
40
20
0
*
*
LNA-1
LNA-2
SCAT7
d
Expression fold change normalized to GAPDH
10
8
6
4
2
0
Ctrl-1
Ctrl-2
LNA1_1
LNA1_2
LNA2_2
LNA2_3
**
FGFR3
FGFR4
p21
e
TGI% relative to Ctrl LNA
100
80
60
40
20
0
***

Supplementary Fig. 8. Silencing of *SCAT7* inhibits tumors growth *in vivo*. **a** Balb/c nude mice showing tumors after eight weeks of subcutaneous injection of 1×10^6 Csh or *SCAT7*-sh1 or *SCAT7*-sh2 A549 cells (n=6 for each group). **b** Immunohistochemistry images of Ki67 staining in xenografts generated from subcutaneous transplantation of Csh or *SCAT7*-sh1 or *SCAT7*-sh2 A549 cells into nude mice showing higher proliferation rate in Csh cells compared to the *SCAT7* KD cells. Scale bars, 100µm. **c** Tumor Growth Inhibition (TGI) of the subcutaneous Balb/c nude A549 xenografts treated with 60 pmol of *SCAT7* LNA-1 and LNA-2, respectively, for a total of four injections (n=5 for each group). Tumors volumes are expressed as mean ± SD, compared to scrambled LNA. **d** Gene expression validation of *SCAT7* targets in the A549 tumors treated with scrambled LNA, *SCAT7*-LNA1, and *SCAT7*-LNA2. Values were expressed as fold change normalized to endogenous GAPDH. **e** Tumor Growth Inhibition (TGI) for the lung patient-derived xenografts (PDX) NSG mice models treated with 100pmol of *SCAT7*-LNA1, for a total of five injections (n=6 for each group). Tumor volumes were expressed as mean ± SD, compared with scrambled LNA.

Figure 6D

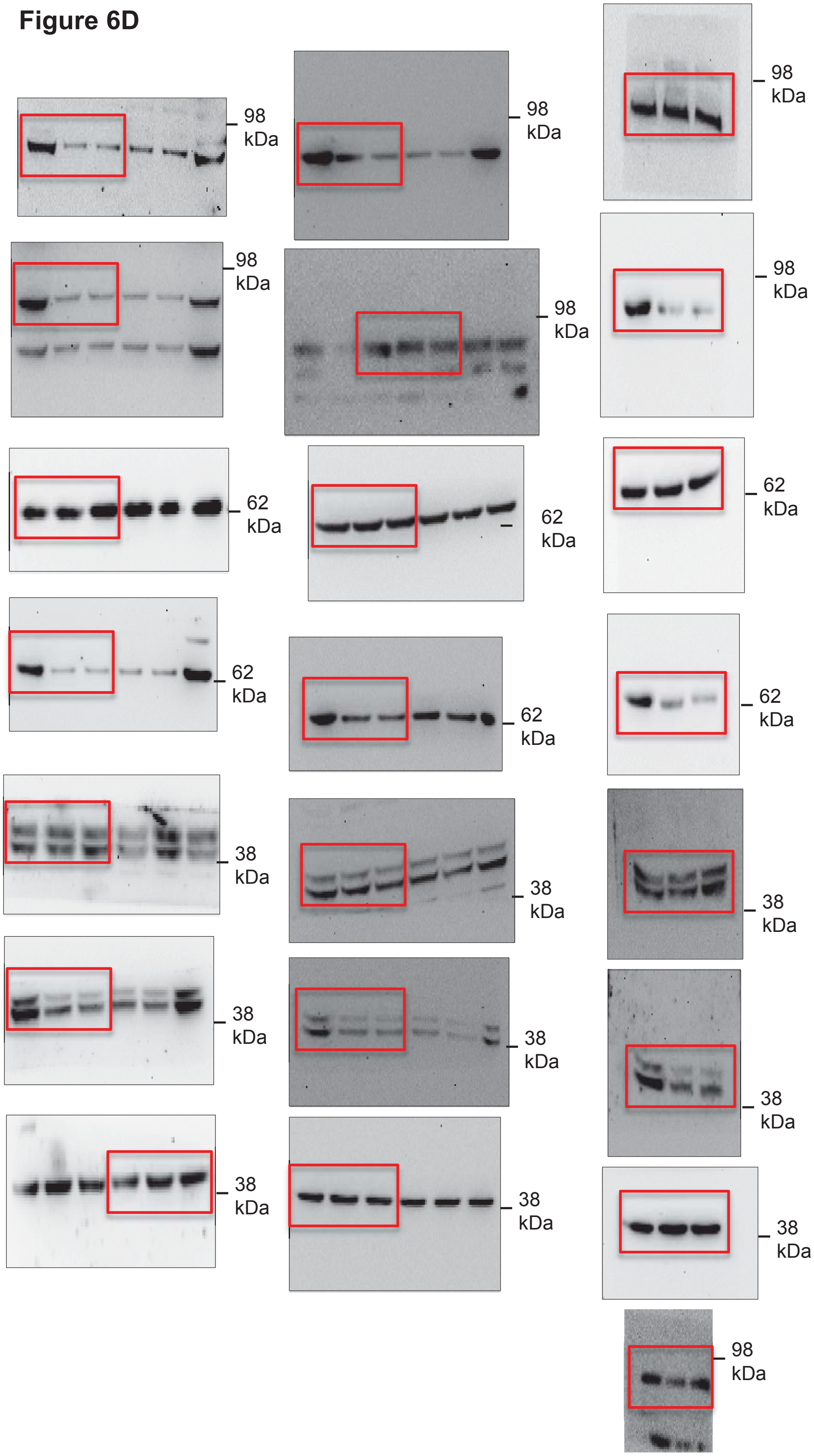

Figure 6E

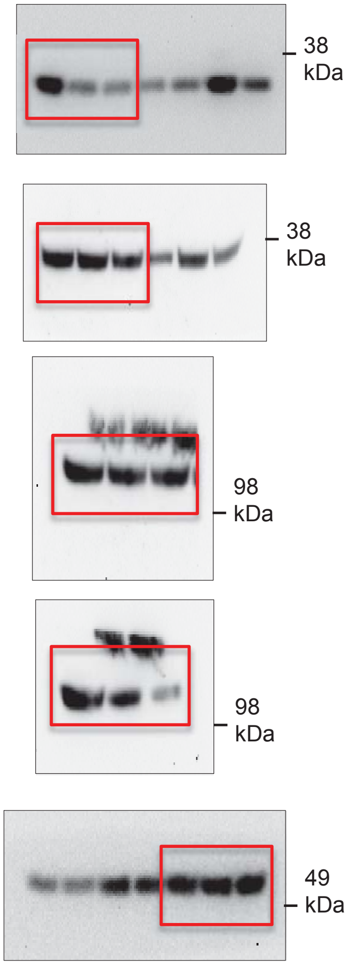

Supplementary Figure 9. Uncropped western blot images. The red sections mark the blot results shown in the indicated figures (figures 6D, 6E).

Figure 7C

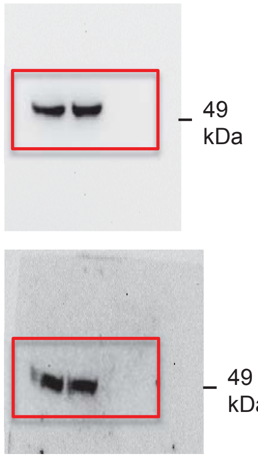

Figure 7G

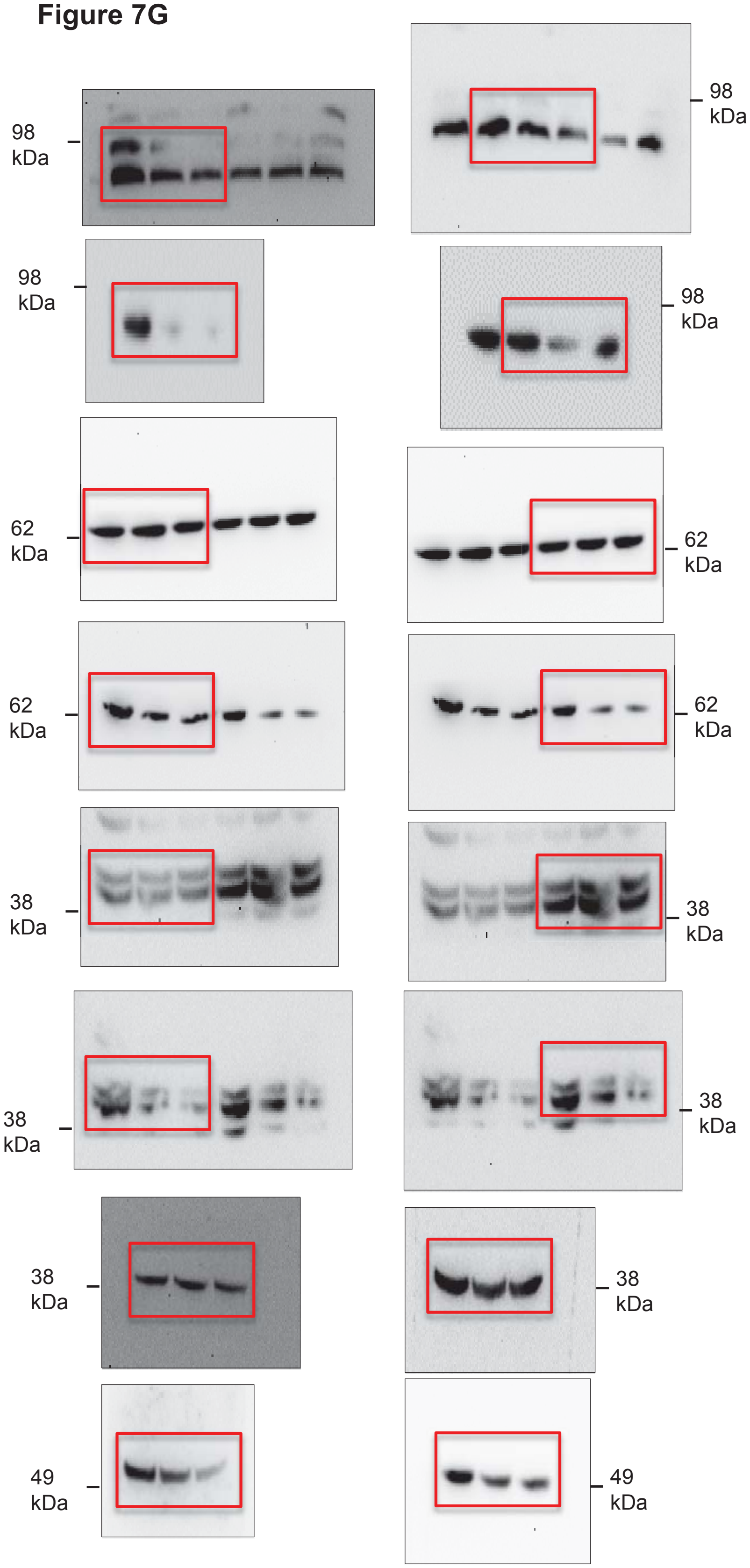

Figure 7L

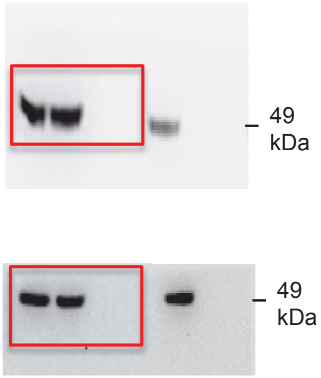

Figure 7O

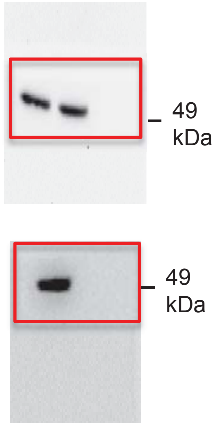

Supplementary Figure 10. Uncropped western blot images. The red sections mark the blot results shown in the indicated figures (figures 7C, 7G, 7L, 7O).
