# Supplementary material for: PAN-cancer analysis of S-phase enriched lncRNAs identifies oncogenic drivers and biomarkers
Source: Nat Commun. 2018 Feb 28;9:883. doi: 10.1038/s41467-018-03265-1 (PMC5830406; doi:10.1038/s41467-018-03265-1)
Supplement: Supplementary file 3 — Description of Additional Supplementary Files [file 41467_2018_3265_MOESM3_ESM.pdf]

**Description of Supplementary Files**

File Name: Supplementary Data 1
Description: Significant and temporally expressed S-phase lncRNAs.

File Name: Supplementary Data 2
Description: Differential expression and clinical analysis of S-phase lncRNAs in different TCGA cancer types.

File Name: Supplementary Data 3
Description: RNA sequencing analysis of SCAT7 by siRNA and shRNA downregulation.

File Name: Supplementary Data 4
Description: List of siRNAs, esiRNAs, shRNAs, LNAs, Oligos and primers used in the study.

File Name: Supplementary Data 5
Description: Details of the antibodies used in the study.
